# Editorial for Special Issue: Metaproteomics

**DOI:** 10.3390/proteomes7010009

**Published:** 2019-03-05

**Authors:** Jana Seifert, Thilo Muth

**Affiliations:** 1 Institute of Animal Science, University of Hohenheim, 70599 Stuttgart, Germany; 2 Bioinformatics Unit (MF 1), Department for Methods Development and Research Infrastructure, Robert Koch Institute, 13353 Berlin, Germany

As the proteome-level counterpart of metagenomics, metaproteomics extends conventional single-organism proteomics and allows researchers to characterize the entire protein complement of complex microbiomes on a large scale [1]. The main objective of metaproteomics is to investigate the key functions provided by multiple organisms that operate the metabolic activity of microbial consortia within an environmental ecosystem or host. It is worth noting that the field has taken an enormous leap forward in the last few years to become more mature and practical nowadays. This progress has been mostly driven by technological advances in mass spectrometry instrumentation, refined experimental setups and protocols, and novel developments of tailored data analysis methods and bioinformatics software. In combination with the increasing amount of genomic information resulting from more cost- and time-efficient DNA sequencing technologies, a better understanding of fundamental processes of microbial consortia has become possible by using metaproteomics. Besides important use cases in environmental studies, the prospects for direct applications of the field are immense in clinical and biotechnological settings and its potential is even increased when metaproteomics is coupled with further meta-omic approaches at the genome, transcriptome, or metabolome levels. Despite many substantial improvements in the recent past, the field still lacks standardized procedures at various layers of the analytical workflow. Indeed, various challenges and problems remain unresolved in the field, among which the most important ones are: (a) the difficulty of choosing representative sampling and protein extraction methods, (b) the issue of selecting appropriate proteomic workflows and experimental settings, and (c) the lack of tools and guidelines for the optimal use of data analysis methods for identifying, annotating, and quantifying proteins to accurately resolve community function and to robustly compare heterogeneous samples across time and space. Therefore, the abovementioned bottlenecks indicate a strong demand for dynamic methodological developments and provision of new validation strategies both in experimental and computational aspects.

The Special Issue “Metaproteomics” from the online journal *Proteomes* welcomed articles describing original research using metaproteomics, as well as metaproteomics in combination with further omics approaches, to identify and describe the key functional players of microbial communities from environmental, human, and animal samples. Furthermore, methodological studies improving metaproteomic workflows and technical descriptions of bioinformatic analysis methods for direct applications in metaproteomics were of interest.

Nowadays, metaproteomics often goes hand in hand with further omics approaches and can, for example, be supported by information at the genome level. Using a metaproteogenomic approach, Palomba et al. [2] investigate luminal and mucosal samples collected from the gastrointestinal tracts of pre-weaned lamb. In their study, they show that analyzing diverse ecological niches along the gastrointestinal tracts can provide microbiota composition and metabolic function that can be further used to study the underlying complex processes of digestion in pre-weaned lamb.

Tröscher-Mußotter et al. [3] investigate intestinal microbiome samples from five different porcine gastrointestinal tract sections using metaproteomics to gain functional knowledge on bacterial groups with a combined analysis of host-specific proteins. Their study presents the first functional investigation of the porcine gastrointestinal tract biology. In this study, the findings show a significant difference between the small and the large intestine of pigs that can be recognized at the bacterial phylogenetic level and by the distribution of functional protein clusters, underlining the physiological differences between these two segments.

Rechenberger et al. [4] present the largest clinical study to date in the field—using metagenomics and metaproteomics, they analyzed 212 fecal microbiome samples from 56 hospitalized acute leukemia patients with multidrug-resistant Enterobacteriaceae gut colonization. Based on their comprehensive data set, the authors discuss the most important issues that affect clinical metaproteomic data analysis, such as incompleteness of public databases and biological variability. To overcome these limitations, they provide guidance to researchers in the field and further suggest which data analysis strategies and study designs need to improved most urgently.

It has become apparent in the past that easily-accessible and well-documented bioinformatics resources are required by researchers conducting metaproteomics studies, particularly in the context of microbiome research and the impact of microbial communities on human health. To meet this need, Blank et al. [5] present a web-based bioinformatics platform for disseminating metaproteomics workflows and software using the Galaxy-P platform. The modular data analysis platform provides different processing steps that are relevant for the metaprotoeomics data analysis, including database generation, peptide spectrum matching, taxonomic annotation, and functional analysis.

Beyond the phylogenetic level, Riffle et al. [6] focus on the functional analysis of a metaproteomics workflow and present MetaGOomics, an online method that automates the quantitative functional analysis of metaproteome data using Gene Ontology (GO) terms. According to the authors, their tool overcomes the issues of classical protein-centric functional analysis, such as size, redundancy, and annotation issues occurring at the protein sequence level.

The studies published in this Special Issue cover the broad field of applications and bioinformatics challenges, which are objectives in the current discourse of the field. The benefit of using a metaproteomics approach to deeply characterize complex microbiome samples is shown. Nevertheless, researchers still hesitate to use this methodology, probably due to the limited accessibility to high-resolution mass spectrometers and the thus-far impaired usability of metaproteomics software tools. This barrier needs to be overcome by establishing robust and easy-to-use bioinformatics tools such as those published in this issue. Future infrastructure projects including investments for machines and data storage will further support a widespread use of metaproteomics.
